# Policy Lessons From Quantitative Modeling of Leprosy

**DOI:** 10.1093/cid/ciy005

**Published:** 2018-06-01

**Authors:** Graham F Medley, David J Blok, Ronald E Crump, T Déirdre Hollingsworth, Alison P Galvani, Martial L Ndeffo-Mbah, Travis C Porco, Jan Hendrik Richardus

**Affiliations:** 1 Department of Global Health and Development, London School of Hygiene and Tropical Medicine, United Kingdom; 2 Department of Public Health, Erasmus MC, University Medical Center Rotterdam, The Netherlands; 3 Zeeman Institute for Systems Biology and Infectious Disease Epidemiology Research, University of Warwick, Coventry; 4 Big Data Institute, Li Ka Shing Centre for Health Information and Discovery, University of Oxford, United Kingdom; 5 Center for Infectious Disease Modeling and Analysis, Yale School of Public Health, New Haven, Connecticut; 6 Francis I. Proctor Foundation for Research in Ophthalmology, University of California, San Francisco

**Keywords:** leprosy, policy, mathematical modeling, elimination, diagnosis

## Abstract

Recent mathematical and statistical modeling of leprosy incidence data provides estimates of the current undiagnosed population and projections of diagnosed cases, as well as ongoing transmission. Furthermore, modeling studies have been used to evaluate the effectiveness of proposed intervention strategies, such as postleprosy exposure prophylaxis and novel diagnostics, relative to current approaches. Such modeling studies have revealed both a slow decline of new cases and a substantial pool of undiagnosed infections. These findings highlight the need for active case detection, particularly targeting leprosy foci, as well as for continued research into innovative accurate, rapid, and cost-effective diagnostics. As leprosy incidence continues to decline, targeted active case detection primarily in foci and connected areas will likely become increasingly important.

Leprosy, caused by the *Mycobacterium leprae* bacterium, persists in at least 122 countries. Globally, about 200000 new leprosy cases, including approximately 18000 in children, are diagnosed every year, with many more thought to be occurring undetected [1, 2]. Delayed diagnosis is problematic from the perspective of disease progression to more serious complications, as well as transmission to others. This, in turn, may lead to neurological impairment and subsequent disabilities characteristic of leprosy [3, 4]. Antibiotic treatment both curbs transmission and halts disease progression, but does not reverse the neurological impairment that has already arisen. Reasons for delayed diagnosis include disregard of early symptoms, difficulties in differential diagnosis (ie, distinguishing between leprosy and other diseases with similar symptoms), and fear of stigma [5].

The epidemiological dynamics of leprosy are driven by the typically prolonged durations between infection and symptom onset, and then between symptom onset and diagnosis [6]. Although data to precisely estimate these periods are scarce, it is clear that the period from exposure/infection to symptom onset ranges from months to decades [6]. The delay between symptom onset and diagnosis is exacerbated by stigma, accessibility of healthcare professionals, and common misdiagnosis, particularly in the initial phase of symptom onset. Most diagnosis is passive (ie, the patient must present to health services), rather than active (ie, the health system actively seeks cases), although many countries implement household contact tracing. Misdiagnosis or delayed diagnosis is compounded by the variability of the clinical presentation upon symptom onset. Bacterial loads typically rise gradually with disease progression, and a large proportion of patients have low bacterial loads (paucibacillary [PB] cases), which are challenging to recognize as leprosy. Conversely, a smaller proportion of patients have very high bacterial loads (multibacillary [MB] cases), and are much more transmissible. Disability increases during the course of infection, so early diagnosis and treatment are crucial to reduce both transmission and sequelae of infection.

In 1991 the World Health Organization (WHO) established the target of eliminating leprosy as a public health problem, defined as a prevalence of <1 per 10000 people, by the year 2000. Although this target was achieved, when averaged across the global population, much higher prevalence persisted in a number of countries [7]. In 2012, a new target was set to achieve “global interruption of leprosy transmission” by 2020. This target was formulated in the London Declaration to support control and elimination of 10 neglected tropical diseases, including leprosy.

Since 2000, the number of annual, newly detected cases has fallen by more than one-half, but transmission is continuing. A lesson learned is that creating quantitative targets can result in perverse outcomes [1, 8]. The 2020 WHO targets were revised in the Global Leprosy Strategy 2016–2020 to become more circumspect—aiming at reducing the global prevalence of grade 2 disability, while maintaining the ultimate goal of transmission elimination [9]. Current interventions are based on prompt diagnosis and treatment. Emphasis is placed on reducing social and legal barriers to early diagnosis, and transition toward active detection of cases in focal high-risk regions.

Leprosy is at a critical point in terms of global and national programs. Its long incubation period creates problems for measuring the impact of immediate policy changes—policy is reacting to changes in transmission that occurred a decade previously, and the impact of changes now will not be seen for a decade. We believe that modeling can play a key role in managing leprosy to zero transmission and zero disability: a leprosy-free world. The purpose of this supplement article is to summarize the key policy-relevant recommendations from recent transmission modeling of leprosy.

## MATHEMATICAL MODELING OF LEPROSY

Mathematical modeling of infectious disease is an efficient and powerful tool for quantifying transmission patterns and for predicting future trends in leprosy diagnoses as well as the potential impact of interventions. Transmission dynamics of infectious diseases are inherently nonlinear. The risks for an individual developing leprosy are determined by the number of cases and the extent of their infectiousness among their contacts, while the incidence in the population is determined by the past history of individual exposure. Consequently, preventing an infection or curing a case also reduces the risk of transmission to the remainder of the population.

The first mathematical models for leprosy were developed by Lechat in the 1970s–1990s [10], from which recent models were adapted [11, 12]. These models provide a mechanistic description of the transmission of leprosy in a population, taking into account the history of infection, the type of leprosy (ie, PB or MB), diagnostic efforts, treatment with multidrug therapy, and relapse rates [13]. Other models explored the potential interactions of leprosy with tuberculosis, both of which are mycobacterial diseases [14].

To explore interventions targeted at contacts of index cases, a stochastic individual-based model was developed that incorporates household transmission dynamics and heterogeneity in susceptibility [15]. This approach explicitly models life histories (ie, birth, household formation, death) and history of infection of individuals. More recently, a sophisticated back-calculation statistical approach was developed to make inferences about the transmission dynamics using information on the time between infection and diagnosis [16].

In 2015, the neglected tropical disease (NTD) Modeling Consortium was set up with funding from the Bill & Melinda Gates Foundation, the Children’s Investment Fund Foundation UK, and the Novartis Foundation to provide modeling analyses of progress toward the London Declaration 2020 targets for 9 neglected tropical diseases. Within the framework of this consortium, the Novartis Foundation provided funding for 3 groups working on leprosy over 2 years. The 3 groups have used different mathematical and statistical modeling approaches, including (1) back-calculation, (2) stochastic compartmental model, (3) the stochastic individual-based model SIMCOLEP, and (4) linear mixed effects regression as a benchmark. Each group further developed and applied its model to answer policy-relevant questions [16–20], in particular whether 2020 targets were likely to be met.

To validate the results of the different models, we have compared the consistency of predictions generated by the respective models [17]. The range of model frameworks employed covers the gamut of quantitative methodologies: multilevel statistical trend models, statistical back-calculation, compartmental transmission models, and fully individualized simulations. All models were fitted to leprosy data obtained from the national Information System for Notifiable Diseases (SINAN) database of 4 Brazilian states with hyper-, high, medium, and low endemicity, respectively. Trends of the new case detection rate (NCDR) were projected until 2040. The different models generated highly consistent mean short-term forecasts over 3 years, with some divergence between longer-term forecasts. Whereas the forecasts of transmission models were constrained by assumptions about infectious disease dynamics, the statistical models do not incorporate such assumptions and, accordingly, exhibited considerably more variability in forecasts. Robust predictions are fundamental to assess progress toward WHO targets and evaluate the effectiveness of strategies to facilitate achieving the targets.

## KEY FINDINGS FROM MATHEMATICAL MODELING

### Feasibility of Global Interruption of Leprosy Transmission

The primary aim of the NTD Modeling Consortium was to provide a quantitative answer to the question of whether global interruption of leprosy transmission could be met by 2020. We focussed on the feasibility of reducing the NCDR to <10 per 100000, which would be indicative of declining transmission. As country-level incidences can mask local dynamics, we focused on state and district levels. Blok et al showed that, given current control, the NCDR is predicted to remain far above the target of 10 per 100000 by 2020 in several states or districts in India, Brazil, and Indonesia [18]. By way of validation, similar results were shown in the model comparison article focusing on 4 states in Brazil [17]. Specifically, long-term predictions of the NCDR trend in Brazil indicated that the target of 10 per 100000 is unlikely to be achieved before 2040 in high- and hyperendemic states. Modeling demonstrated that global interruption of leprosy transmission is unfeasible within 2 decades [17, 18].

An important concern that the modeling has highlighted is the imprecision of the concept of “interruption of transmission.” This can be interpreted as the complete cessation of transmission, which is highly unlikely if there are any extant infections. Alternatively, it could correspond to the reduction in the reproduction number, *R*_*0*_, below 1, the threshold below which each case transmits to <1 new case on average. The latter target is much more feasible, and would eventually achieve the global eradication of leprosy. Nonetheless, the eradication process would play out over many, many infection cycles; as each cycle lasts many years, the timely eradication of leprosy is highly unlikely without further, novel interventions, especially given the presence of nonhuman reservoirs.

The major impediment for achieving interruption of transmission is the pool of undiagnosed symptomatic and asymptomatic leprosy cases, which contribute to the transmission of *M. leprae*. Modeling has shown that the number of undiagnosed symptomatic and asymptomatic cases is approximately twice the number of diagnosed cases [21], although the exact ratio of symptomatic to asymptomatic cases is likely to vary according to the epidemiology (eg, whether transmission is increasing or decreasing) and diagnostic effort (ie, duration between onset of symptoms and diagnosis). This substantial pool of infected persons, still contributing to the transmission of *M. leprae*, must be addressed if leprosy is to be eliminated and, ultimately, eradicated.

### Geographical Variation

The model results emphasize the geographical variability in both current trends and future predictions. Under status quo interventions, regions with lower incidence are predicted to reach the 10 per 100000 threshold within a few years, whereas those with higher incidence are predicted to have only a small chance of reaching this threshold within 20 years. This pattern is also reflected at a state level (eg, Brazilian states) and may apply at smaller spatial scales [22]. The general spatial distribution of leprosy is characterized by a shrinking to “islands” of high incidence in which transmission is perpetuated. These foci contribute disproportionately to ongoing transmission and will be particularly challenging to control with current interventions. On the other hand, a more limited geographic distribution of leprosy will become increasingly feasible to consider mass interventions, such as chemoprophylaxis, targeted on these areas to achieve further reduction both geographically and epidemiologically.

A limitation of the current models is that they do not include the influence of human movement and migration. A recent study reported leprosy prevalence as high as 10% from a sample of people recruited from a bus station in Brazil [23]. The individuals sampled were self-selected following an awareness and recruitment campaign. This study highlights 2 important points. First, as predicted by models, there is a considerable burden of undiagnosed cases. Second, populations moving (especially on buses) may be at elevated risk of infection than the general population: Approximately one-third of diagnosed cases did not reside in the recruitment catchment area, and approximately one-half were residing in a state different from their birth. Both leprosy and use of public transportation are likely to be positively related to poverty [24]. The assumptions of mathematical models are typically that population size is constant, such that turnover is limited to equal rates of birth and death. The statistical approaches that have been used predict the future number of cases that will contribute to the data stream without geographic stratification. However, these Brazilian data suggest that many leprosy cases may not be diagnosed where they are infected.

### Impact of Additional Control Strategies and Tools

Modeling has highlighted the need for a shift in the current leprosy control strategies to improve the likelihood of achieving an NCDR <10 per 100000 in a reasonable time frame, and ultimately interrupting transmission [17, 18, 21]. To develop effective intervention strategies, a better understanding of *M. leprae* transmission and its risk factors is still needed [25]. Models can play an important role in testing assumptions about the transmission of *M. leprae.* For example, the stochastic individual-based model SIMCOLEP has been used to assess the different mechanisms of susceptibility, ranging from random to genetic mechanisms [15]. More importantly, models can be used to identify new entry points for strategies, and to evaluate the efficacy and cost-effectiveness of additional control strategies.

**Figure 1.**
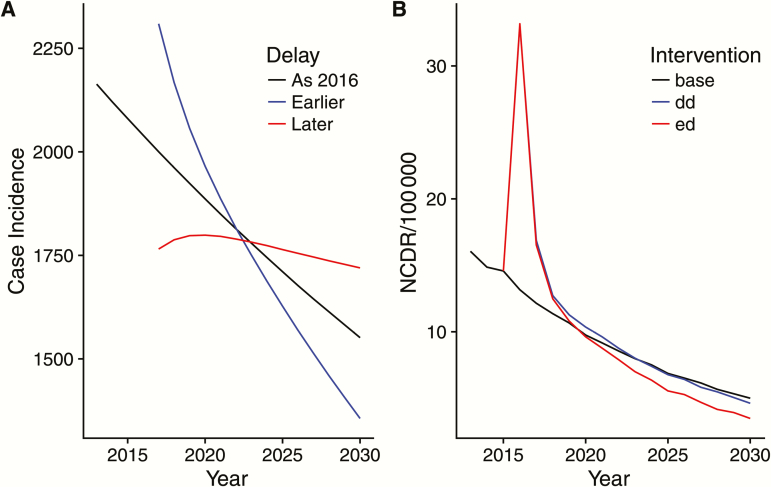
The predicted incidence of leprosy with changing diagnostic delays. *A*, Output from back-calculation showing how increasing delay (red) and decreasing delay (blue) alter current and future incidence (mean predicted values fitted to Ceara, Brazil). *B*, Output from SIMCOLEP showing how decreasing the passive case detection delay by 2 years (blue line) and adding a diagnostic test to detect asymptomatic cases among household contacts of an index patient (red line) alters current and future new case detection rate in leprosy in Amazonas, Brazil. SIMCOLEP predictions of the baseline scenario (black line) were based on the model quantification in Blok et al [17]. Abbreviations: dd, detection delay (shorter); ed, early diagnosis including detected asymptomatic cases; NCDR, new case detection rate.

Universally, model results underscore the importance of more rapid diagnosis and treatment, preferably during the asymptomatic stage, to further reduce the NCDR of leprosy [16, 21]. The back-calculation analysis highlighted that earlier diagnosis will initially increase the number of diagnoses, but this will eventually fall due to the success of the intervention, whereas slower diagnosis has the opposite effect, that is, exacerbating future incidence (Figure 1A). The SIMCOLEP results echo this pattern (Figure 1B). A reduction in passive case detection delay by 2 years will first increase the NCDR as previously undiagnosed symptomatic cases are detected, but will then decline. Adding a test to detect asymptomatic leprosy cases among household contacts of a patient, followed by appropriate treatment, will further decrease the NCDR.

It is important to note that for leprosy, underlying changes in case incidence are masked in different ways in surveillance data. If there is a slowing in case detection, then this is unlikely to be detectable from the surveillance data for many years (Figure 1, blue line), meaning that a large burden of undetected cases grows [1]. This suggests that alternative measures of the success of a program should be reported alongside the NCDR, such as the age of cases, the self-reported time from onset of symptoms to detection, or some estimate of the likely date of exposure for the subset of cases for which this can be identified.

Given the importance of decreasing the time to diagnosis, there are a number of tools being developed to reduce this time and prevent transmission from identified cases [5]. Innovative ways to prevent leprosy include administering chemoprophylaxis or immunoprophylaxis to contacts of patients with newly diagnosed leprosy, and earlier diagnosis through screening with diagnostic tools to identify leprosy patients. A modeling study concerning Pará State in Brazil showed that administering chemoprophylaxis to household contacts, in addition to the current control (ie, passive case detection and treatment, and household contact tracing), would further lower the NCDR and would accelerate achieving a level of 10 per 100000 [20]. Efficiency would also be improved by a diagnostic test that is able to detect asymptomatic leprosy cases. Model results showed that the NCDR could be reduced by approximately 50%–80% in the long run, depending on the sensitivity of the test and leprosy endemicity. Even sensitivity as low as 50% could achieve a substantial reduction (Blok et al, unpublished data).

### Future Steps and Data Needs

Mathematical modeling can be a useful tool for informing the design of programs and policies to control and eliminate leprosy. However, modeling needs data, and there is a need to ensure accuracy, veracity, and completeness of reported data. Open access to annual national and subnational leprosy data, including data about the details and implementation coverage of the control program, is essential. Also, improvements in granularity of case data would be informative, including specifying age groups, household size, and information about the context (eg, geographical location, migration history). Additional measures of program efficacy are needed to optimize control strategies, given that the NCDR is a function of the effort expended to find them. Data pertinent to program efficacy include time from symptom onset to diagnosis, program coverage, and compliance rates of active case finding. Studies about stigma as well as legal and cultural issues might inform the design of multifaceted programs that address interdependent social and epidemiological challenges.

Mathematical models have thus far focused on predicting the NCDR and number of new cases of leprosy, whereas disability due to leprosy has received relatively little attention. As the level of grade 2 disability, especially among children, is also an indication of the ongoing transmission and delayed diagnosis, there is a need to take grade 2 disability into account more formally. Models could inform policy makers about the future burden of disease and the impact of new strategies on the incidence of disability. To accurately incorporate these factors, additional data are needed about the disability burden and the time between onset of symptoms and onset of grade 2 disability.

Finally, when moving toward zero transmission, the NCDR increasingly becomes an unreliable indicator of reduction in transmission, given the confounding that arises from the interdependent relationship between diagnostic effort. A more accurate measure of transmission elimination would be, for example, the absence of leprosy cases among children [26]. For mathematical models to be informative, accurate reporting of cases among children and further new tools to monitor progress toward zero transmission are needed. These tools remain to be developed.

## CONCLUSIONS

The succession of control, local elimination, and global eradication of leprosy will—at a minimum—require many decades. Persons infected today may take 20 years to develop clinical symptoms. A diagnostic that permitted diagnosis of asymptomatic cases would be a “game-changer.”

Incidence is highly aggregated in some areas, and migration and movement are important issues in many of these populations. It may be, for example, that migrant cases from high-incidence population are maintaining transmission in the low-transmission areas. Targeting intervention in such foci might achieve disproportionate returns. Generally, the prolonged duration between infection acquisition and symptom onset, combined with human migration, means that cases will not necessarily be diagnosed where they are infected. These geographical and temporal gaps are barriers to contact-tracing efforts aimed at efficiently identifying other cases.

Novel approaches to prophylactic treatment and diagnosis that enable the detection of asymptomatic infection will likely be fundamental to achieve elimination and, ultimately, eradication. Mathematical modeling can be used to design clinical trials of new diagnostics and to evaluate the cost-effectiveness of implementation programs. As leprosy transmission becomes increasingly clustered, the opportunity for mass interventions becomes more feasible. Mathematical modeling can be used to determine optimal thresholds of local incidence and geographical distribution to trigger shifting between targeted and mass interventions within foci. The criteria for these triggers should incorporate data-driven relationships between improvements in diagnosis as screening becomes more effective and rates of case reporting increase.

Current modeling is limited by data availability and knowledge about the disease. With limited knowledge about transmission, simple models are usually as effective as more complex ones. There is a need for more detailed data to enable models to be refined, particularly in terms of individual-level, epidemiological data relating to age and geography. Such data will enable the tailoring of intervention design to different settings between which optimal strategies likely vary. In order to continue to control leprosy, and to eventually eliminate and eradicate it, we need continued investments in data collection, modeling analyses, and diagnostic innovations.

## Notes

***Disclaimer.*** The views, opinions, assumptions, or any other information set out in this article are solely those of the authors and should not be attributed to the funders or any person connected with the funders.

***Financial support.*** This work was supported by the Bill & Melinda Gates Foundation in partnership with the Task Force for Global Health through the NTD Modelling Consortium (grant number OPP1053230) and the Novartis Foundation.

***Supplement sponsorship.*** This article appears as part of the supplement “Reaching the 2020 Goals for Nine Neglected Tropical Diseases,” sponsored by the NTD Modelling Consortium.

***Potential conflicts of interest.*** All authors: No reported conflicts of interest. All authors have submitted the ICMJE Form for Disclosure of Potential Conflicts of Interest. Conflicts that the editors consider relevant to the content of the manuscript have been disclosed.
